# Recent Techniques to Improve Amorphous Dispersion Performance with Quality Design, Physicochemical Monitoring, Molecular Simulation, and Machine Learning

**DOI:** 10.3390/pharmaceutics17101249

**Published:** 2025-09-24

**Authors:** Hari Prasad Bhatta, Hyo-Kyung Han, Ravi Maharjan, Seong Hoon Jeong

**Affiliations:** 1 College of Pharmacy, Dongguk University, Goyang 10326, Republic of Korea; 2 College of Pharmacy and Yonsei Institute of Pharmaceutical Sciences, Yonsei University, Incheon 21983, Republic of Korea

**Keywords:** amorphous solid dispersion, stability, thermodynamics, machine learning, simulation, drug–polymer interaction, drug release

## Abstract

Amorphous solid dispersions (ASDs) represent a promising formulation strategy for improving the solubility and bioavailability of poorly water-soluble drugs, a major challenge in pharmaceutical development. This review provides a comprehensive analysis of the physicochemical principles underlying ASD stability, with a focus on drug–polymer miscibility, molecular mobility, and thermodynamic properties. The main manufacturing techniques including hot-melt extrusion, spray drying, and KinetiSol^®^ dispersing are discussed for their impact on formulation homogeneity and scalability. Recent advances in excipient selection, molecular modeling, and in silico predictive approaches have transformed ASD design, reducing dependence on traditional trial-and-error methods. Furthermore, machine learning and artificial intelligence (AI)-based computational platforms are reshaping formulation strategies by enabling accurate predictions of drug–polymer interactions and physical stability. Advanced characterization methods such as solid-state NMR, IR, and dielectric spectroscopy provide valuable insights into phase separation and recrystallization. Despite these technological innovations, ensuring long-term stability and maintaining supersaturation remain significant challenges for ASDs. Integrated formulation design frameworks, including PBPK modeling and accelerated stability testing, offer potential solutions to address these issues. Future research should emphasize interdisciplinary collaboration, leveraging computational advancements together with experimental validation to refine formulation strategies and accelerate clinical translation. The scientists can unlock the full therapeutic potential with emerging technologies and a data-driven approach.

## 1. Introduction

There are several advanced techniques to improve drug release performance of poorly soluble drugs. Notable ones are glycosylation [1], specific group engagement [2], co-crystallization [3], complexation, and more intriguing methods [4]. Amongst them amorphous solid dispersion (ASD) has been one of the preferred strategy formulations for poorly soluble drugs classified under biopharmaceutical classification system (BCS) Class II and Class IV [5]. The bioavailability of poorly soluble drugs primarily depends on their solubility and dissolution profile in biological fluids [6]. The drug crystals are converted into an amorphous form, which enhances its solubility, dissolution rate, and bioavailability [7]. In ASD systems, the crystalline drug is incorporated into a suitable polymer carrier, resulting in transformation into an amorphous state, which eliminates the need to break the crystal lattice [8]. Consequently, the amorphous form of many poorly soluble drugs attains substantially higher apparent solubility and a markedly faster dissolution rate [9]. However, producing the amorphous form requires significant energy input, rendering it susceptible to phase separation and recrystallization during manufacturing and storage [10].

The molecular mobility of the amorphous state increases upon exposure to ambient temperature and humidity, promoting recrystallization [11]. The crystalline drugs possess a well-defined, ordered structure with strong intermolecular bonds, conferring superior stability and predictable physicochemical behavior such as solubility, drug release, and melting point depression [12]. The intermolecular arrangement within crystals, extending in all directions, results in single or polycrystalline forms at the micron scale [13]. Molecules can adopt different conformations within the lattice, a phenomenon known as polymorphism [14]. Therefore, different polymorphic forms of the same drug exhibit distinct physicochemical properties. Different polymorphic forms exhibit distinct melting points. Thermal energy input drives structural reorganization toward more thermodynamically stable configurations, while accelerated cooling kinetics allow preservation of metastable crystalline arrangements before complete liquefaction occurs [15].

The amorphous form lacks long-range order and defined shape. Its molecular arrangement is unpredictable, leading to high intermolecular energy and metastability [16]. The amorphous forms possess higher free energy compared to their crystalline counterparts due to increased Gibbs free energy, which translates to greater apparent solubility and is advantageous for poorly soluble drugs. However, the amorphous form is thermodynamically unstable and tends to recrystallize over time or under stress conditions [17]. The absence of long-range order in the amorphous state results in short-range molecular interactions that promote clustering and nucleus formation [18,19].

This review investigates the physical stability of ASDs from a physicochemical perspective, considering thermodynamic, kinetic, and environmental aspects. The thermodynamic factors influencing ASD stability include drug solubility in the polymer, phase separation, drug–polymer compatibility, glass transition temperature, and drug–polymer interactions [20]. The kinetic factor associated with stability can estimate the molecular mobility, nucleus formation, and nucleus growth [21]. Environmental factors, such as temperature and humidity, can affect the physical stability of the amorphous form through both thermodynamic and kinetic mechanisms. Environmental conditions may induce polymorphic transformations, altering drug behavior [22]. Additional factors, including suboptimal formulation component selection, thermal and manufacturing stresses, and increased molecular mobility, can promote crystal precipitation, coarsening, and aging, ultimately diminishing dissolution rate and bioavailability [23]. Moreover, this review focuses especially on the recent development tools related to multiscale simulations, artificial intelligence (AI)—machine learning (ML), computation tools, and molecular modeling. Additionally, the impact of the tools is also discussed to perform efficiently high-throughput screening, predict ASD optimization, improve evaluation, and assess risk management [24,25,26,27,28,29,30,31]. The stable ASD products require careful selection of formulation components, manufacturing procedures, process parameters, and packaging [32].

ASDs are commonly classified into three generations: first generation (amorphous drug only), second generation (polymeric carrier), and third generation (amorphous carrier with surfactant) [33]. They are typically prepared by solvent evaporation or heat-congealing. Among these, spray drying, hot-melt extrusion, and KinetiSol^®^ are widely used for industrial-scale production. Currently, 48 drug products containing ASDs, representing 36 unique amorphous drugs, have been approved by the U.S. FDA and are commercially available. These dosage forms include tablets, capsules, and granules. Table 1 summarizes commercially available drug products manufactured using ASD technology. The spray drying and hot-melt extrusion (HME) are most common industrial methods. The historical trend shows an increase in approval of ASDs since the early 2000s. The major polymers are the industry workhorses, most notably HPMCAS and PVP-VA64. Most ASDs are formulated as tablets [34].

Solid-state characterization techniques are employed to investigate the thermodynamic and kinetic properties of the amorphous form. The crystallinity in ASDs is typically assessed by differential scanning calorimetry (DSC), Raman spectroscopy, infrared (IR) spectroscopy, and powder X-ray diffraction (PXRD) [35]. Advanced imaging technologies such as nano-tomography and terahertz spectroscopy enable investigation of intermolecular interactions between polymer and drug at the submicron scale [36,37]. These innovative characterization methods facilitate prediction of dissolution properties, elucidation of recrystallization patterns, and evaluation of stability outcomes [11,38,39,40]. In addition, classic theories, molecular modeling, and machine learning approaches for assessing physical stability factors are introduced and discussed [41].

## 2. Effects of Material and Process Attributes

ASDs can be prepared using the drug alone via different preparation methods; however, such dispersions tend to recrystallize rapidly. Therefore, excipients or stabilizers are incorporated prior to processing to induce the “parachute effect.” Hydrophilic polymers are commonly used as excipients. The choice of polymer plays a crucial role in ASD stability [42]. These polymeric carriers are classified into distinct groups according to their chemical structure and physicochemical properties for use in dosage forms. Table 2 summarizes polymers commonly employed in ASD development. Among these, cellulose polymers derived from natural plant cellulose are widely used to enhance drug solubility, stabilize amorphous forms, and control drug release [43,44,45,46]. Vinyl polymers, synthesized from vinyl pyrrolidone or vinyl caprolactam monomers, exhibit diverse physicochemical properties depending on side chain chemistry [47]. Polymethacrylate polymers, derived from acrylic acid esters (acrylates) and methacrylic acid esters (methacrylate), are used as adhesives, enteric film formers, sustained-release agents, moisture protectants, and for pH-dependent solubility [48,49]. Polyvinyl acetate phthalate (PVAP) is a nonionic produced by polymerization and dissolves at basic pH [50]. Polyacrylic acid (PAA) is a synthetic polymer made from acrylic acid monomers and is notable for its water solubility and high-water absorption capacity [51]. Polyethylene glycol/polyethylene oxide is used as a crosslinker with carriers in ASD formulations [52,53].

**Figure 1:**
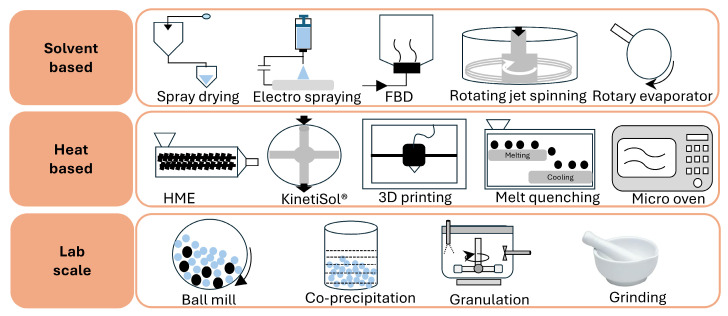
Schematic diagram of major ASD preparation methods: solvent- and heat-based manufacturing techniques along with other methods, i.e., ball mill, co-precipitation, granulation, and grinding [adopted with permission from Elsevier [60]].

Figure 1 provides an overview of principal and advanced manufacturing techniques. The stability of the amorphous form is influenced by process parameters such as speed, temperature, flow rate, drying rate, and residual solvent, all of which affect molecular mobility and crystallization risk [57,58]. In the case of solvents, even when removed after post-processing, they can significantly impact the final product’s properties and stability by modulating drug–polymer interactions, evaporation rates, and the potential for phase separation and recrystallization. Solvents differ in miscibility and interaction strength, thereby affecting ASD stability and dissolution. Additionally, evaporation kinetics vary among solvents. Table 3 presents solvent selection criteria, which depend on boiling point, solubility, and toxicity, in accordance with ICH guidelines [59].

### Quality Design and Process Parameter Tools

**Figure 2:**
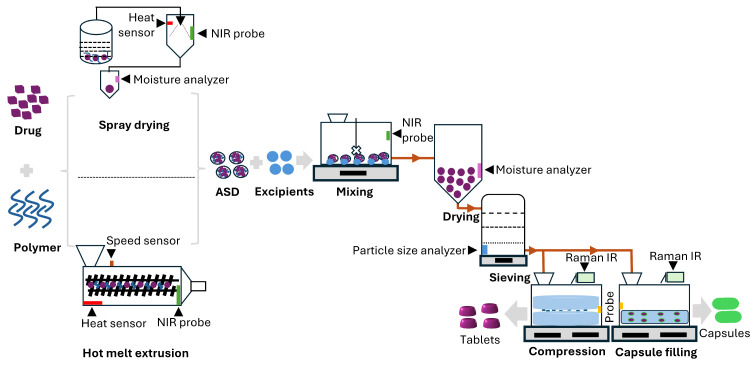
Schematic diagram depicting the manufacturing process of pharmaceutical solid dosage forms of ASD (mixing, drying, sieving, compression or capsule filling) by applying combined QbD and PAT tools. The spray drying and HME processes are two major manufacturing types discussed.

The quality design and process parameters encompass quality by design (QbD) and process analytical technology (PAT) approaches to ensure product quality throughout manufacturing. QbD incorporates various process parameters, including critical quality attributes (CQAs), critical material attributes (CMAs), critical process parameters (CPPs), and design space (DS), all of which play significant roles in ASD quality. CQAs identify key attributes that affect ASD performance, such as solubility, stability, and dissolution rate. CMAs inform product design and understanding. CPPs define manufacturing parameters that influence CQAs, such as temperature, mixing speed, and solvent evaporation rate, while DS establishes the CPP range that consistently yields high-quality ASDs [61,62]. PAT emphasizes real-time monitoring (on-line, in-line, or at-line) and control of manufacturing processes. Figure 2 illustrates in-line and on-line monitoring with near-infrared (NIR) and Raman spectroscopy, used to assess crystalline state, particle size, and uniformity during production. Such monitoring enhances understanding and control, ensuring consistent quality and reducing waste [63,64,65,66].

The integration of QbD and PAT offers a robust framework for ASD manufacturing. QbD establishes the design space and CQAs, while PAT maintains process control within this space through real-time monitoring. This constructive collaboration enhances product quality, lowers production costs, and accelerates the time to market for new formulations. Such approaches mark a departure from conventional trial-and-error methods toward more systematic, data-driven strategies. For example, the optimization of extrusion processes for ASDs of piroxicam with Kollidon^®^ VA64 using a QbD methodology involved in-line UV–Visible spectroscopy to monitor CQAs in real time during manufacturing, demonstrating that PAT provides a reliable tool for tracking and managing ASD quality during HME [67].

The physicochemical properties of polymers significantly influence CQAs, which include the nature of the polymer type, molecular weight, polydispersity, glass transition temperature (*T_g_*), particle size, mechanical properties, and chemical stability [68]. It is important to understand how the property of polymer influences the process parameters and affects the final product’s likelihood of crystallization in the final product [69]. The monitoring of physical stability during storage, handling, and dissolution testing is critical in early formulation development [70]. To guarantee the therapeutic efficacy of the final product remains a primary regulatory objective in ASD development. This objective is addressed through the application of diverse analytical techniques such as DSC, polarized light microscopy (PLM), Raman spectroscopy, infrared (IR) spectroscopy, dielectric spectroscopy, and powder X-ray diffraction (PXRD) at each stage of development [71].

## 3. Physical Stability of Amorphous Solid Dispersion

Many amorphous drug forms exhibit instability during preparation and storage, which presents a major challenge for formulation scientists [60,72]. Amorphous forms lack a defined structure and exist in a metastable state characterized by increased entropy, enthalpy, free energy, and volume. Consequently, these forms have an inherent thermodynamic tendency to crystallize. However, when functional groups capable of hydrogen bonding are present, stabilizing drug–polymer interactions can occur, increasing the energetic barrier to crystallization [73]. At room temperature, the molecules in a solid dispersion vibrate at their relative position and do not display diffusive mobility. Over long-term storage, the amorphous state gradually evolves toward thermodynamic equilibrium at the storage temperature, altering its physical structure. The drug loading affects solid-state saturation solubility under storage conditions, and over time, this may result in phase separation and recrystallization [74]. The amorphous–amorphous phase separation (AAPS) is characterized by the formation of distinct drug-rich and polymer-rich amorphous domains within the ASD matrix, which initially consisted of a molecularly mixed system. The drug-rich phase is generally more susceptible to crystallization due to limited polymer inhibition, whereas the polymer-rich phase exhibits greater stability because of the uniform distribution of polymer within the ASD matrix [75]. The following sections focus on key factors influencing ASD stability, emphasizing thermodynamic and kinetic properties as well as environmental influences.

### 3.1. Thermodynamic Factors on Physical Stability

#### 3.1.1. Solubility of Drug in Polymer

The solubility of a drug in a polymer matrix is a critical determinant of ASD stability and performance. It governs the extent to which the drug remains molecularly dispersed, thereby preventing recrystallization and improving bioavailability [73]. When drug concentration surpasses its saturated solubility, the solid dispersion exhibits elevated chemical potential within the metastable system, increasing the likelihood of recrystallization. Thermodynamically, equilibrium between crystalline and dissolved (or dispersed) drug corresponds to the formation of a saturated solution [76]. In this context, the polymer acts as the solvent, and the crystalline drug as the solute; the saturated solubility of the drug in the polymer can be calculated using the solid–liquid equilibrium equation [24].

(1) $$\mathrm{In}ᵪ\mathrm{drug}\;=\frac{∆\mathrm{Hfus}}{\mathrm{RTm}}\;(1-\frac{T_{m}}{T})-\;\mathrm{In}ᵧ\mathrm{drug}$$

where ᵪdrug is the mole fraction solubility of the drug in the polymer, ∆Hfus is the drug’s melting enthalpy, R is the gas constant, *T_m_* is the drug’s melting point, *T* is the two-phase equilibrium temperature, and ᵧdrug is the drug’s activity coefficient. These thermodynamic parameters can be determined from DSC data. Thus, drug solubility in the polymer at various temperatures can be calculated using activity coefficients of *ᵧdrug*. The activity coefficient is estimated using the extended Hansen model as shown below:

(2) $${\mathrm{In}}_{\gamma }\mathrm{drug}=\frac{V\;\mathrm{drug}}{\mathrm{RT}\left\{(\delta_{d}^{\mathrm{drug}}-\bar{\delta }{\;d)}^{2}+0.25\left[{(\delta }_{\rho }^{\mathrm{drug}}-{\bar{\delta }}_{\rho }{)}^{2}+{(\delta }_{h}^{\mathrm{drug}}-{\bar{\delta }}_{h}{)}^{2}\right]\right\}}+\;\mathrm{In}\frac{\mathrm{drug}}{\bar{V}}+1\frac{V_{\mathrm{drug}}}{\bar{V}}$$

where Vdrug is the molar volume of the drug, δdrug denotes the solubility parameter for the drug, and $\bar{\delta }$ denotes the solubility parameter for the combined system (weighted average), with subscripts *d*, *ρ*, and *h* indicating dispersion, polar, and hydrogen bonding forces, respectively, and $\bar{V}$ indicating the mixed volume. The calculations proceed as follows:

(3) $$\delta =\sum_{k1}^{n}\emptyset k\delta k$$

where ∅k is the volume fraction of component *K*.

(4) $$\emptyset \kappa =\frac{\chi_{k}}{\bar{V}}V_{k}$$

where $\chi_{k}$ and $V_{k}$ are the mole fraction and molar volume of component *K*, respectively.

(5) $$V\;k=\frac{\mathrm{Mk}}{\rho k}$$

where Mk and ρk are the molecular weight and density of component *K*, respectively.

(6) $$\bar{V\;}=\sum_{k=1}^{n}x_{k}V_{k}$$

The exact solubility limit is found by extending this line to the point where no heat change occurs (zero melting energy). This zero point means all drug has dissolved completely—no crystals remain. This method works because only undissolved drug crystals produce measurable melting heat. In practice, combinations of drug and polymer at different concentrations are examined by DSC. The solubility of cinnarizine in Soluplus^®^ at different drug concentrations was determined using phase diagrams to identify the thermodynamically stable region of the binary system [77]. As drug loading increases, melting enthalpy rises, but solubility in the polymer remains unchanged, indicating a supersaturated and thermodynamically unstable dispersion. The reduced drug loading enables molecular dispersion, forming a solid solution that supports high dissolution rates and improved drug bioavailability [78].

#### 3.1.2. Phase Separation

Phase separation is a critical process in which an initially homogeneous drug–polymer mixture separates into two distinct phases—typically drug-rich and polymer-rich regions—significantly influencing performance, stability, dissolution, and bioavailability. This phenomenon commonly arises when drug loading exceeds the miscibility threshold within the polymer matrix, particularly during storage [79]. The amorphous–amorphous phase separation (AAPS) can be triggered by factors such as temperature, humidity, drug loading, and intrinsic drug–polymer miscibility. The AAPS in ASDs can be characterized using various analytical techniques, including DSC, IR spectroscopy, nuclear magnetic resonance (NMR), confocal fluorescence microscopy (CFM), scanning electron microscopy (SEM), PXRD, and dielectric spectroscopy [80,81].

#### 3.1.3. Compatibility of Drug–Polymer Combinations

Drug–polymer compatibility is essential for maintaining ASD stability and efficacy. The metastable form supports the maximum drug loading within the polymer and possesses high intermolecular energy; exceeding this limit induces phase separation and ultimately leads to drug crystallization. The Flory–Huggins lattice theory provides a means to estimate drug–polymer compatibility [82].

(7) $$\;∆G_{\mathrm{mix}}=\;\mathrm{RT}\left[\emptyset \;\mathrm{In}\emptyset +\frac{\left(1-\;\phi \right)\;\mathrm{In}(1-\phi )}{m}+\chi \emptyset \;(1-\emptyset )\right]$$

where R is the gas constant, T the absolute temperature of the drug, ∅ is the volume fraction, m is the number of lattice sites occupied by the polymer chain, and X is the drug–polymer interaction parameter. The parameter m is calculated as follows:

(8) $$m=\frac{M_{\mathrm{Polymer}}/\rho_{\mathrm{polymer}}}{M_{\mathrm{drug}}/\rho_{\mathrm{drug}}}$$

where M and ρ are the molecular weight and density of the polymer or drug, respectively. The interaction parameter χ can be estimated from solubility parameters [83]:

(9) $$\chi \approx 0.34+\frac{\nu_{\mathrm{drug}}}{\mathrm{RT}}(\delta_{\mathrm{drug}}\;-{\;\delta }_{\mathrm{polymer}}{)}^{2}$$

where $\nu_{\mathrm{drug}}$ is the drug’s molar volume, and $\delta_{\mathrm{drug}}$ and $\delta_{\mathrm{polymer}}$ are the solubility parameters of the drug and polymer, respectively. The spinodal equation is derived by setting the second derivative of the Flory–Huggins free energy ($∆G_{\mathrm{mix}})$ in Equation (7) to zero and obtaining the spinodal equation:

(10) $$\mathrm{Ts}\;=\;\frac{2V\;(\delta_{\mathrm{drug}\;}-\;\delta_{\mathrm{polymer}}{)}^{2}}{R}x\;\frac{1}{\frac{1}{\phi \;}\;+\;\frac{1}{\;m\;(1\;-\;\phi )}}$$

The region below the spinodal line denotes where phase separation spontaneously occurs, resulting in drug crystallization. The drug–polymer compatibility can be evaluated by the extent of the area bounded by the spinodal region [68]. The accuracy of the spinodal line depends on the precision of the interaction parameter, as this parameter dictates intermolecular interactions and mixing between drug and polymer [69,70].

#### 3.1.4. Glass Transition Temperature

Glass transition temperature (*T_g_*) is a key thermophysical property of amorphous materials. It defines the temperature at which a solid transitions to a rubbery, fluid-like state. The amorphous state’s characteristic *T_g_* makes it a crucial parameter for assessing ASD stability [84]. When homogeneous and heterogeneous intermolecular forces are equivalent, the drug and polymer form an ideal mixture, and the amorphous system displays a single *T_g_*. Under these conditions, the *T_g_* of the ASD follows the Fox equation [85]:

(11) $$\frac{1}{\mathrm{Tg}}=\frac{W_{1}}{{\mathrm{Tg}}_{1}}+\frac{W_{2}}{{\mathrm{Tg}}_{2}}$$

where $T_{g_{1}}$ and $T_{g_{2}}$ are the actual glass transition temperatures of the components, $W_{1}$ and $W_{2}$ are the mass fractions of the components, and *T_g_* is the expected glass transition temperature. The intermolecular forces between like and unlike components are equivalent when the actual *T_g_* is close to the expected value, suggesting a uniform distribution of the drug in polymer. If the observed *T_g_* exceeds the predicted value, then the intermolecular forces between like molecules are weaker than those between unlike molecules. Conversely, if the actual *T_g_* is lower than predicted, the intermolecular forces among like molecules are stronger than those among unlike molecules. A large negative deviation between observed and predicted *T_g_* suggests that self-interactions of the drug molecules are stronger than drug–polymer interactions, favoring aggregation into large amorphous clusters and phase separation.

**Figure 3:**
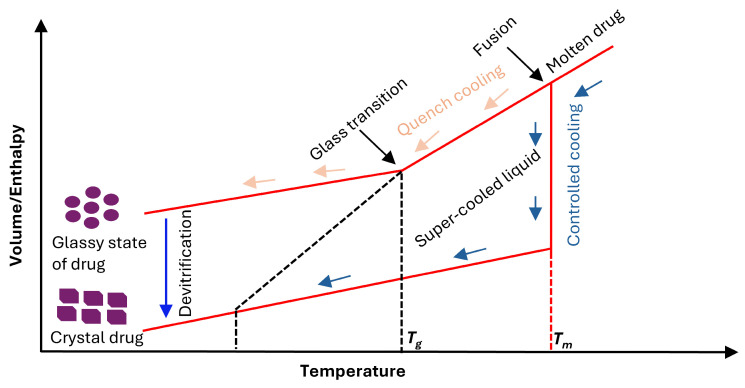
Schematic diagram on the relationship between the temperature and volume/enthalpy of the drug to form crystalline or amorphous forms. Red line represents the equilibrium state of drug showing the volume/enthalpy changes with temperature for each phase. Blue arrows show the slow cooling path where the molten drug slowly cooled. Orange arrows are a rapid cooling, where molten drug cooled quickly and bypassed the crystallization at *T_m_* and continued as a liquid into the supercooled region. The blue line (devitrification) is the transformation of metastable glassy state back to stable crystalline state. Diagram not to scale [adopted with permission from Elsevier [87]].

Figure 3 illustrates the conversion from crystalline to amorphous forms and subsequent reconversion at room temperature. When a drug mixed with polymer forms dispersion, the new *T_g_* of the mixture generally ends up between the individual *T_g_* values of the drug and polymer. This elevated *T_g_* effectively makes the kinetic barrier for crystallization. Melting temperature (*T_m_*) is the temperature at which crystalline solid undergoes first-order phase transition to become a liquid upon heating. On slow cooling, molten drug turns crystal. *T_g_* is the temperature at which super cooled liquid transitions into a rigid glass state upon cooling. Fusion is the sharp vertical increase in volume/enthalpy at *T_m_* as the crystal drug melts into a molten drug and this requires an input of energy [86,87]. This principle motivates the “*T_g_* 50 °C rule” where molecular mobility in an amorphous solid decreases markedly and becomes negligible when the temperature is approximately 50 °C below its *T_g_*. This empirical rule is widely applied in material science, particularly for polymers and pharmaceuticals. For instance, the physical stability of 52 amorphous drugs was investigated by using the DSC. A support vector machine (SVM) model was used to predict physical stability. The drugs were classified into two groups: Class II, 18 drugs that crystallize upon heating, and Class III, 34 drugs that remain amorphous. The drugs were stored for 12 h at 20 °C above and below the *T_g_*. The results showed that 14 compounds from Class II crystallized when stored above the *T_g_*, whereas excepting one molecule, the Class III remained amorphous [88]. Therefore, polymers with high *T_g_* are selected for ASD development, as they enhance both drug and polymer solubility. This approach improves bioavailability and maintains drug stability by preventing phase separation and crystallization. In addition, polymeric carriers function as stabilizers for the amorphous form during prolonged storage [89].

#### 3.1.5. Drug–Polymer Interaction

Drug molecules interact with polymers through hydrogen bonding, van der Waals forces, ionic interactions, electrostatic interactions, or hydrophobic interactions, all of which play key roles in ASD physical stability. The hydrogen bonding notably affects the stability of nifedipine with various polymers, with the strength of hydrogen bonding, structural relaxation time, and physical stability following the order: polyvinylpyrrolidone (PVP) > hydroxypropyl methylcellulose succinate (HPMCAS) > polyacrylic acid (PAA) [90]. The strongest drug–polymer interactions were observed in PVP-based ASDs, effectively inhibiting amorphous drug crystallization [91]. The introduction of hydrophobic segments into water-soluble polymers can shield drug–polymer interactions from atmospheric moisture, thereby reducing ASD hygroscopicity and drug supersaturation [92]. Pea protein isolate, a novel plant-derived polymer, can replace animal proteins in pharmaceuticals, improving drug precipitation inhibition and stability during dissolution [93,94]. The acidic polymer PAA, when combined with clofazimine to form drug–polymer salts, creates strong ionic interactions, and enhances crystallization resistance [95]. Additionally, halogen bonding influences dissolution profiles by lowering the binding energy between drug and polymer, increasing drug solubility [96]. The drug–polymer interactions reduce molecular mobility, enhance physical stability, and can be assessed by IR spectroscopy, Raman spectroscopy, and solid-state NMR [97,98].

### 3.2. Kinetic Factors on Physical Stability

#### 3.2.1. Molecular Mobility

Molecular mobility is a critical factor governing ASD crystallization. In the amorphous state, drug molecules exhibit a strong tendency to migrate and rearrange, promoting crystallization, especially as mobility increases. Amorphous molecules undergo continuous thermal vibrations, leading to the glass transition, or “global mobility.” These molecular motions are categorized as α-relaxation, primarily involving rotation and translation of functional groups in both drug and polymer. When the ambient temperature exceeds *T_g_*, molecular vibrations intensify, and α-relaxation becomes the dominant driver of amorphous material recrystallization (*T* > *T_g_*). For example, broadband dielectric spectroscopy has been used to evaluate the molecular mobility of itraconazole in ASDs containing PVP or HPMCAS [99]. The dielectric spectra revealed that α-relaxation times for itraconazole increased with HPMCAS, while PVP had minimal effect. An isothermal crystallization study confirmed that HPMCAS inhibited crystallization more effectively than PVP, suggesting a strong relationship between α-relaxation and crystallization kinetics [100].

The local molecular motion in amorphous systems, termed β-relaxation (secondary relaxation or local mobility), involves bond rotation or whole-molecule motion. When the ambient temperature is below *T_g_*, the molecular system is in a rigid or “frozen” state (*T* < *T_g_*), favoring nucleation and recrystallization. The olanzapine–malic acid film system shows, secondary relaxation in the intermediate frequency range of the dielectric spectrum may result from intermolecular interactions [101]. The models such as Adam–Gibbs–Vogel and Vogel–Tammann–Fulcher equations are commonly applied to describe the structural relaxation of drug–polymer combinations for predicting long-term physical stability [102]. In the supercooled state, molecular mobility, represented as the reciprocal of the relaxation time τ, exhibits pronounced temperature dependence, as described by the VTF equation [103]:

(12) $$\tau \;=\;\tau_{0}\exp\;(\frac{{\mathrm{DT}}_{0}}{T_{f\;}\;-{\;T}_{0}})$$

β-relaxation follows Arrhenius temperature dependence, as shown below:

(13) $$\tau \;=\;\tau_{0}\exp(\frac{{Ea}_{\beta }}{\left(\mathrm{RT}\right)})$$

where τ is the average relaxation time, $\tau_{0}$ is the relaxation time constant under ideal environments, D is the intensity constant, $T_{f}$ is the virtual temperature, and $T_{0}$ is the temperature at which molecular mobility becomes zero. R is the gas constant, and $E_{\alpha \beta }$ is the activation energy for the β-relaxation process, which is typically much lower than the energy required for α-relaxation. Notably, β-relaxation involves longer structural relaxation times and requires less energy for molecular mobility.

Thus, when the temperature is below *T_g_*, crystallization of solid dispersions is suppressed, increasing physical stability. For example, crosslinking ketoconazole–PAA amorphous dispersions with PVA progressively reduced molecular mobility and improved physical stability as crosslinker content increased [104].

#### 3.2.2. Nucleus Formation

Nucleus formation generally initiates with molecular mobility within the amorphous matrix, allowing molecules to aggregate and form stable clusters. These clusters function as nuclei, triggering the transition from the amorphous to crystalline state. According to classical nucleation theory (CNT), the formation of a crystal nucleus via phase interaction is described as follows [105]:

(14) $$∆F\;=\;∆F_{\nu }+\;\Delta F_{I}$$

where ∆F is the change in free energy during nucleus formation, $∆F_{\nu }$ is the change in chemical potential due to volume change, and $\Delta F_{I}$ is the change in interfacial free energy. The rate of crystal nucleus formation can be calculated as follows [106]:

(15) J = B exp (−∆G/k T)

where J is the rate of nuclei formation per unit time and volume, B is the kinetic pre-factor, ∆G is the free energy change for the formation of a nucleus at its critical radius, *k* is the Boltzmann constant, and T is the absolute temperature. The kinetic pre-factor B is calculated as follows [107]:

(16) $$B\;=\;\frac{\nu P^{2}}{{k\;T}^{2}}\;\sqrt{2\sigma }/\pi m$$

where P is the vapor pressure, σ is the surface tension, m is the molecular weight, and ν is the molecular volume. The factor ∆G can be calculated as follows [108]:

(17) $$∆G\;=\;16\pi v^{2}r_{\mathrm{ns}}^{3}/3(k\;T\;\mathrm{In}(s){)}^{2}$$

where v is the mobility of molecules or atoms at the crystal interface, $r_{\mathrm{ns}}$ is the interfacial energy per unit area, and *s* is the supersaturation. Substituting Equations (16) and (17) into (15) yields the following [109]:

(18) $$B\;=\frac{\nu P^{2}}{{k\;T}^{2}}\;\sqrt{2\sigma }/\pi m\;\exp\;\frac{-16\pi v^{2}\;r_{\mathrm{ns}}^{3}}{3(k\;T{)}^{3}(\mathrm{Ins}{)}^{2}}$$

This equation demonstrates that increasing surface tension (σ) slows nucleation, while increasing supersaturation accelerates it. Thus, selecting polymers with good compatibility can reduce nucleation rates and recrystallization. Taylor et al. reported that strong hydrogen bond donors in PAA increased the nucleation rates of amorphous acetaminophen, whereas HPMCAS, which contains weaker hydrogen bond donors and acceptors, most effectively inhibited nucleation. Their study found no direct correlation between nucleation rates and easily identifiable system properties, such as drug–polymer interactions or *T_g_* [110]. The polyethylene terephthalate has been employed as a heteronucleant in the melt crystallization of acetaminophen in PEG to form crystalline solid dispersions. The acetaminophen exhibited two polymorphic forms, form I and form II. The coating of acetaminophen with heteronucleants inhibited polymorphic transitions by 10% compared to the uncoated substrate [111]. A faster cooling rate during manufacturing increases amorphous yield and stability in highly drug-loaded ASDs, ultimately facilitating nucleus formation [112].

#### 3.2.3. Growth of Nucleus

**Figure 4:**
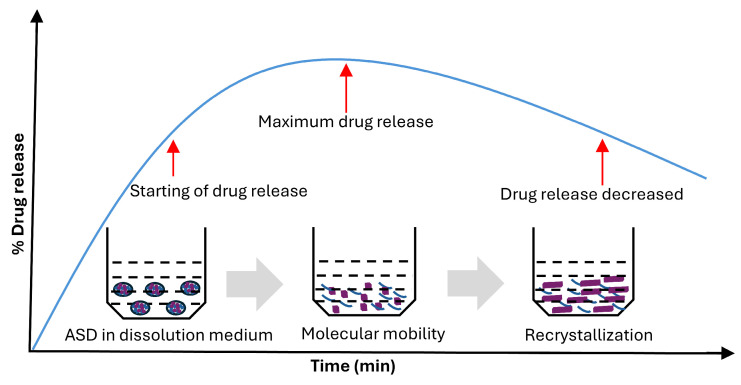
Schematic representation of molecular mobility and drug recrystallization over time. Once the solid dispersion contacts the dissolution medium and starts the drug release, after a certain time, drug release decreases and solid dispersion changes to the crystalline form. Diagram not to scale [adopted with permission from Elsevier [87]].

Crystal growth commences immediately after nucleus formation, according to crystal growth and diffusion theory. This process comprises two main steps: drug molecules diffuse from the dispersion medium and accumulate on the surface of the crystal nucleus, after which they are incorporated into the crystal lattice, releasing the heat of crystallization [7]. The crystal growth rate is typically represented by the rate of increase in crystal radius, calculated as follows [85]:

(19) $$\frac{d_{r}}{d_{t}}\;=\;[D_{v}N_{A}/(r\;+D/k_{+})](C\;-\;C_{\mathrm{eq}})$$

where *D* represents the diffusion coefficient of drug molecules, $k_{+}$ is the surface coagulation factor, *N_A_* is the Avogadro’s constant. The term $\left(C\;-\;C_{\mathrm{eq}}\right)$ signifies the concentration gradient between the drug in its carrier and the drug molecules that have not attached to the crystal surface. Equation (19) shows that the mechanism controlling crystal growth depends on the crystal’s size. When $r\;\gg \;D/k_{+}$, the crystal growth is limited by diffusion. In contrast, when $r\;\ll \;D/k_{+}$, the growth is primarily governed by the surface aggregation factor. As the crystal expands, the viscosity, molecular migration rate, and $(C\;-\;C_{\mathrm{eq}})$ of the system change; thus, Equation (19) has inherent limitations [113]. Figure 4 illustrates that an increased crystal growth rate negatively impacts dissolution performance by reducing solubility and promoting crystal seeding, which leads to de-supersaturation [114]. The ASD of itraconazole with PVP-K12 and HPMCAS demonstrated that systems containing soluble polymers exhibit a higher tendency to crystallize than those with insoluble polymers, resulting in a sharp decline in drug concentration in the dissolution medium [115]. The nuclei formation and growth are frequently discussed in the protein crystallization methods while some literature can be found even for small molecules [116].

### 3.3. Environmental Factors on Physical Stability

The stability of ASDs is influenced by environmental factors that indirectly affect both thermodynamic and kinetic stability. Key environmental factors include temperature, humidity, storage conditions, and light exposure. Molecular mobility is intricately linked to temperature; as temperature approaches *T_g_*, molecular mobility increases, which can significantly impact ASD stability. Enhanced molecular mobility raises the likelihood of phase separation and recrystallization [117,118]. Humidity in the environment is absorbed by the ASD, disrupting hydrogen bonding between the drug and polymer and thereby reducing *T_g_*. For instance, absorption of 1.0% moisture by an amorphous system can lower its *T_g_* by 10 °C. When a hydrophobic polymer (Eudragit^®^ EPO) and a hydrophilic polymer (PVP-VA64) are co-extruded, phase separation occurs due to their immiscibility. After co-extrusion with the drug, a solid dispersion is formed with a microstructure resembling an emulsion. In this system, the hydrophobic phase contains a small amount of drug as the continuous phase, while the hydrophilic phase contains a larger quantity of drug as the dispersed phase. The hydrophilic (dispersed) phase is coated by the hydrophobic (continuous) phase, which inhibits moisture penetration [119].

A 20% drug-loaded cinnarizine-Soluplus^®^ ASD was prepared via HME. The samples stored at 40 °C and 75% humidity, and at 60 °C and 94% humidity, exhibited similar effects on physical stability. Thus, both temperature and humidity had comparable impacts on the stability of the dispersion system [113]. In another study, individual felodipine, carbamazepine, celecoxib, and fenofibrate were formulated with Eudragit^®^ EPO using the HME method. It was found that high humidity (75% RH) induced greater crystallization than elevated temperature (40 °C). This effect was observed at both low (10%, *w*/*w*) and high (70%, *w*/*w*) drug loadings, underscoring the importance of humidity control in ASD storage and formulation [120].

## 4. Molecular Simulation and Statistical Methods

When a drug is distributed within a carrier, various intermolecular forces play significant roles in preventing drug molecule aggregation. These forces include hydrogen bonding, acid-base or ionic interactions, dipole–dipole forces, and van der Waals interactions. Such interactions reduce self-association, enhance drug–carrier binding strength, and contribute to long-term physical stability [121,122,123]. This challenge can be addressed using various molecular techniques. The quantum mechanic (QM), molecular mechanic (MM), and molecular dynamic (MD) approaches are applied to investigate intermolecular interactions, molecular mobility, solubility, and stability. Table 4 summarizes the application of MDs, QMs, and docking studies in ASD systems to simulate molecular mobility and intermolecular interactions between drug and polymer.

### 4.1. Quantum Mechanics (QMs)

QM methods, such as density functional theory (DFT), offer valuable insights into non-bonding interactions between drugs and carriers. These forces influence stability and binding affinity via molecular complexes and interaction energies. The modeling of non-bonding interactions has emphasized DFT as an effective computational approach for predicting interaction energies and identifying hydrogen bonding pairs. Periodic DFT computations are increasingly used as a modeling tool to analyze solid-state pharmaceutical compounds. The combined DFT/MD approaches are employed to investigate drug–polymer molecular behavior by utilizing electronic structure information [150,151]. Large, structurally realistic systems are challenging to model accurately; however, molecular modeling can predict a wider range of properties and chemical behaviors. The strong drug–polymer interactions influence miscibility and stability [152]. The polymer and formulation screening strategy play an important role in assessing the solubility and stability of ASDs. For instance, positively charged (cationic) drugs like propranolol hydrochloride and diphenhydramine hydrochloride interact with ionic carriers like Eudragit^®^ L100 and Eudragit^®^ L100-55 [153]. These carriers are considered in their monomeric form with different configurations and polymers, both with and without the presence of chloride (Cl^−^) ion in the complex. The DFT calculation revealed that the most significant stabilizing force is the formation of a hydrogen bond between the drug’s amine group and the polymer’s ester and hydroxyl groups. The most optimal interaction occurs between the drug’s amine and the polymer’s carbonyl groups. The presence of chloride (from the drug’s salt form) can disrupt the weaker hydrogen bond (involving hydroxyl group) but does not affect the more critical hydrogen bonding of the amine group. The findings suggest that the strongest interaction occurs between the amine group of propranolol or diphenhydramine and the carbonyl group of Eudragit^®^ L100 or Eudragit^®^ L100-55 polymers. These computer-based predictions were confirmed with physical laboratory methods, especially NMR and X-ray photoelectron spectroscopy. The DFT computation calculated that the most stabilizing interaction occurs between the tertiary amine and carboxyl groups (20 to 28 kcal/mol) with binding energy of 5 to 8 kcal/mol for each additional hydrogen bond [133]. The interactions between the amine group of drug molecules (cetirizine HCl and verapamil HCl) and the carboxyl group of polymers (Eudragit^®^ L100 and Eudragit^®^ L100-55) showed the strongest binding after extrusion, indicating high binding energy and the formation of a more stable amorphous form [134]. The partial charge analysis of clofazimine and HPMC phthalate revealed strong donor and acceptor sites, with a simplified model system using acetic acid as a structural substitute for hypromellose phthalate carboxylic groups. The calculations and spectroscopic studies suggested that ion pair complex formation is a key factor for drug–carrier miscibility [154]. PVP inhibited crystallization of resveratrol–PVP and griseofulvin–PVP for storage stability. DFT calculations revealed greater drug–carrier interaction strength in the resveratrol–PVP complex than in the griseofulvin–PVP system. The stronger interaction energies led to higher stability, as evidenced by the sustained high dissolution rate of the PVP–resveratrol system after storage, compared to the lower dissolution rate of the griseofulvin–PVP ASD [155]. The QMs insights were used to screen polymer compatibility and process related factors in the advanced drug delivery system [148].

### 4.2. Molecular Mechanics (MMs) and Molecular Dynamics (MDs)

MD simulations require parameterized system components for molecular mechanic (MM) force fields, which compute total energy by summing empirical potential energy functions [156]. Commonly applied force fields include condensed phase optimized molecular potentials for atomistic simulation studies (COMPASSs) [157], polymer consistent force field (PCFF) [158], chemistry at Harvard macromolecular mechanics (CHARMMs) [159], assisted model building with energy refinement (AMBER) [160], and optimized potential for simulations of liquids, crystals, proteins, and polymers (OPLS) [161]. The force fields represent energy as a function of atomic positions, with potential energy divided into two categories: (a) bonded interactions, describing bond lengths, angles, and torsions, and (b) non-bonded interactions, accounting for Coulombic and van der Waals forces. Chemical phenomena are essential for predicting drug–polymer miscibility and molecular mobility, such as flexible chain motion or hydrogen bond formation. These interactions vary during the simulation and can be averaged over its duration or across multiple runs from different initial conditions [162,163]. One of the limitations of these simulations is that it may not be possible to analyze all the chemical surfaces. The prediction of specific chemical surfaces largely depends on the initial model selection and parameter condition settings.

Classical molecular dynamics (MDs) adhere to Newtonian mechanics, monitoring atomic positions, velocities, accelerations, forces, and energies at each simulation step. At any given time, total energy depends on atomic positions, which are updated iteratively at each step based on the forces acting on each atom. Recent advances in computation have made MD simulations of amorphous drug–drug [164,165], drug–micelle [166], polymer–polymer [167,168,169,170], polymer–membrane [171], and polymer–plasticizer [172] systems routine in the scientific literature.

The MD simulations of ASD systems model drug interactions with polymer carriers. The MM-based docking and MD simulations predict a range of phenomena and properties. The docking simulations investigate the drug–carrier interactions, while MD simulations assess drug–carrier miscibility based on solubility and Flory–Huggins interaction parameters. These simulations have shown that ASDs form through hydration and dissolution mechanisms. The drug–excipient solubility parameters can be computed via MD simulation [124]. The miscibility of artemisinin in PEG and PVP was determined by MD simulation at 298 K, revealing a solubility parameter difference (∆δ) of 0.08 for artemisinin–PVP, 0.57 for artemisinin–PEG, and 0.15 for artemisinin–PVP–PEG (50:50 *w*/*w*), indicating drug miscibility with the polymer blend [127]. The MD simulation has been used to investigate the drug loading efficiency of gemcitabine at different chitosan concentrations [135]. The MD simulations of copolymer–curcumin systems monitored conformational adjustments and distance changes between components. Initially, the drug and polymer were separated by a distance beyond the van der Waals interaction range; after 100 picoseconds (ps) of MD simulation, continuous interactions formed binding sites on the polymer surface [126]. The hydrogen bonding occurred between the hydroxyl group of the polymer and the chlorine group of lafutidine. The MD simulations showed that lafutidine with Soluplus^®^ and Lutrol^®^ exhibited the lowest energy and strongest bonding interactions, confirming stable ASD formulation [131]. The hydroxyl group of the polymer and the chlorine group of posaconazole established hydrogen bonds. According to MD simulation, the most stable dispersion had the greatest number of bonding contacts and the lowest energy [132]. MD simulations have clarified interactions between ritonavir and Lutrol^®^, revealing that the oxyethylene moiety in Lutrol^®^ interacts with the hydrophobic group of ritonavir, thereby increasing ritonavir solubility in the molten phase and forming a stable solution [22].

### 4.3. Docking Studies of Drugs in Polymer Carriers

Docking is commonly employed to generate favorable preliminary binding conformations that are subsequently refined through molecular dynamic (MD) simulations. This approach provides an algorithmic method for rapid sampling and scoring of drug–polymer carrier complexes. The intermolecular interaction of lumefantrine with Soluplus^®^, Lutrol^®^ F127, Lutrol^®^ F68, and PEG 4000 revealed strong hydrogen bonding between polymer hydroxyl and carbonyl groups and the drug’s chlorine and amine groups [128]. The docking system was also used to determine the binding energy of the tautomeric di-keto and keto-enol forms of curcumin with monomer and dimer units of Eudragit^®^ EPO. The di-keto form exhibited higher binding energy than the keto-enol form, involving van der Waals forces, Coulombic interactions, and hydrogen bonding [173]. MD simulations of clonazepam, ibuprofen, fenofibrate, and alprazolam with polymers such as PVP-VA64, HPMC, and Eudragit^®^ EPO were conducted to assess miscibility and intermolecular interactions. The results showed that ibuprofen/PVP-VA64, ibuprofen/Eudragit^®^ EPO, ibuprofen/HPMC, clonazepam/PVP-VA64, clonazepam/HPMC, fenofibrate/PVP-VA64, fenofibrate/Eudragit^®^ EPO, alprazolam/PVP-VA64, alprazolam/Eudragit^®^ EPO, and alprazolam/HPMC combinations were miscible. However, hydrogen bond analysis indicated that only ibuprofen/PVP-VA64 and ibuprofen/Eudragit^®^ EPO formed strong hydrogen bonds that stabilized solid dispersions, while the other drug/polymer pairs exhibited weak or no hydrogen bonding [140].

A systematic docking simulation using full-length polymers evaluated the anchoring ability of cyclosporin A across various polymer chain lengths: short (~7 nm), medium (13–14 nm), and long (~20 nm) for L/D polylactide, chitosan, polyglycolic acid, PEG, and cellulose. Scientists generated one million molecular models for each polymer type and chain length, showing the interaction between a fixed drug cyclosporin A and flexible polymer. The results showed that chitosan and cellulose had the most favorable interactions with cyclosporin A [129]. The miscibility of ibuprofen and carbamazepine with Soluplus^®^/PEG was assessed using the Hoftyzer–Van Krevelen and Hildebrand solubility parameters. The molecular docking images showed uniform distribution of drugs and polymers in ternary systems. The carbamazepine–Soluplus^®^/PEG system had a more negative binding affinity (−6.2 to −6.7 kcal/mol) than the ibuprofen–Soluplus^®^/PEG system (−5.3 to −5.5 kcal/mol), indicating stronger interactions between carbamazepine and Soluplus^®^/PEG. The calculated solubility parameters and DSC experiments confirmed the miscibility of each ternary system. Additionally, FT-IR spectroscopy revealed strong hydrogen bonding among the carbamazepine primary amine, carbonyl, and amide groups, as identified by docking and MD simulations [143].

The dynamic simulations of naproxen, diclofenac sodium, dimethyl fumarate, and omeprazole with polymers (HPMCAS, HPMCP, and Eudragit^®^ L100) were performed to elucidate the molecular interactions between delayed-release drugs and enteric polymeric excipients. The optimal API–polymer pairs identified were naproxen–Eudragit^®^ L100, diclofenac sodium–HPMCP, dimethyl fumarate–HPMCAS, and omeprazole–HPMCAS. All APIs formed hydrogen bonds with polymeric excipients. However, as API loading increased, API–polymer interactions decreased, resulting in higher API mobility and accelerated release. The increased temperature further enhanced API mobility, leading to faster release [174]. The solubility and Flory–Huggins (FH) interaction parameters for the amorphous indomethacin–PVP system were evaluated by varying drug candidates, polymers, and water content in each MD simulation [130]. While differences in the solubility parameters of indomethacin and PVP predicted borderline miscibility (δIMC-PVP = 6.5 MPa1/2), FH interaction parameters predicted complete miscibility (XIMC-PVP = −0.61).

Similarly, FH parameters were calculated for felodipine ASD with HMPC and water. The hydrogen bonding between felodipine and HMPC promoted miscibility, although these bonds were disrupted by added water [141]. The formation of hydrogen bonds between Lutrol^®^ F68 and two carbamazepine molecules, investigated by MD simulation, indicated a high tendency for carbamazepine aggregation and phase separation [136]. MD simulations showed that binding affinity and solvation free energy can inhibit the crystallization of telaprevir with carboxylate-containing polymers, suggesting that MDs can serve as a predictive tool for screening suitable polymers [137].

Another study examined indomethacin mixed with PEG and polylactic acid (PLA) polymers, focusing on ASD formation via simulated annealing, API–polymer miscibility using MD-predicted FH interaction parameters, and polymer carrier encapsulation efficiency [139]. The in silico ASD screening predicted drug–polymer compatibility for solubility enhancement [142]. The MD simulation showed that drug release increased with higher PEG concentrations in modified PLA carriers [144]. MD simulations also identified suitable carriers for olmesartan medoxomil, enhancing dissolution with PVP-VA64 and Soluplus^®^. The interactions between drug and polymer were investigated using atomistic MD simulations [145].

Solubility and FH interaction parameters were determined for tacrine with chitosan and polybutylcyanoacrylate (PBCA) polymers. Tacrine showed greater miscibility with PBCA by both methods, and MD simulations indicated that longer polymer chains yielded higher interaction energies. Computational and experimental studies of simvastatin and PVP predicted miscibility, dynamic Hansen solubility, and FH parameters, as confirmed by DSC experiments [138]. For rivaroxaban with Soluplus^®^, specific molecular interactions and shrinkage led to a drug-rich amorphous phase, resulting in recrystallization under high humidity [147]. The molecular dynamics indicated weak hydrogen bonding between erlotinib HCl and PVP or PEG individually, but a combination of PVP and PEG enabled hydrogen bond formation and enhanced molecular interactions [149].

## 5. Machine Learning for Better Performance

Machine learning (ML) plays a transformative role in the preparation and stabilization of ASDs, improving drug solubility and bioavailability while reducing experimental workload. ML, a branch of artificial intelligence (AI), has driven rapid advances in silico drug development over the past decade. ML uncovers complex, nonlinear relationships between input parameters and target features. It leverages large experimental datasets and data-driven supervised algorithms for drug formulation optimization. ML algorithms such as transfer learning, one-shot, zero-shot, and Bayesian-based optimization have gained popularity for enhancing model performance with sparse data [175,176]. Deep learning (DL), a subset of ML, is typically represented by artificial neural networks (ANNs) that emulate neural connectivity in the brain. In ANNs, nodes are interconnected directly or indirectly through multiple layers. The information enters via the input layer, is processed by hidden layers, and reaches the output layer. ANNs are particularly effective at identifying complex, nonlinear relationships between input and output variables.

ANNs are increasingly applied in drug development and process optimization. They play major roles in modern machine learning for predicting and enhancing the composition, long-term stability, and dissolution rate of ASDs. In addition, other ML algorithms include genetic algorithms (GAs), multiple linear regression (MLR), logistic regression (LR), decision trees (DTs), random forest (RF), k-nearest neighbor (kNN), Naïve Bayes (NB), and light gradient boosting (LGBM) [177,178]. Statistical learning models predict properties and phenomena occurring between drug molecules and carriers, especially when experimental data for model training is limited. Despite such constraints, ML models designed to predict amorphous API properties provide valuable insights for rational ASD and formulation development. Multiple linear regression (MLR) models have been used to predict the long-term physical stability of the amorphous form for 25 poorly soluble drug candidates by employing physiochemical properties derived from two-dimensional structures, as well as measured thermodynamic and kinetic solid properties such as fusion, relaxation time, and configurational free energy. Features relevant for predicting the amorphous behavior of APIs may also play a significant role in predicting drug–carrier ASD systems [179]. Table 5 summarizes recent ML predictions for physicochemical properties, stability, and formulation strategies of ASDs. The dissolution kinetic was predicted for 616 dissolution profiles by using the LightGBM model. The results indicate that the improved LightGBM model exhibited a notable enhancement in predictive performance, with MAE decreasing from 0.124 to 0.111 [29]. CNNs were used to find the impact of the fiber diameter of electrospun on the solubility of ASDs. The experiment used 161 images, and this model identified the spherical shape with 93.2% accuracy [180]. The solubility and phase behavior of drug–polymer mixtures was predicted by the DNNs. This model utilized 499 solubility data points with the molecular descriptor for hydrogen bond interaction and found *r*^2^ 0.92 [181]. An RF model was used to predict the *T_g_* of 50 formulations by using inputs of hydrophilic backbone methylation, hydrophilic feed fraction, and hydrophobic backbone methylation. The result revealed that RF accurately predicted *T*_g_ with an average *r*^2^ > 0.83 [182]. The amorphization and chemical stability of ASDs during manufacturing was predicted for 760 formulations which were prepared from the combination of different drugs (*n* = 49) and excipient types. The result found that ECFP-LightGBM was the best model to predict amorphization with an accuracy of 92.8% [183]. The crystalline and amorphous content in formulation containing the rivaroxaban with Soluplus^®^ was predicted using the ANN, PLS, and PCR models. The 30 experiments were performed and it was found that the root mean squared error of prediction was 0.86 (crystalline) and 2.14 (amorphous). The ANN model found a more powerful and sophisticated regression than the PLS and PCR models [147]. The long-term physical stability of solid dispersions was determined by using ANN, SVM, RF, DT, LightGBM, kNN, NB, and DNN. These inputs that these models utilized were drug loading ratio, polymer molecular weight, drug properties, environmental conditions, preparation method, and temperature. The 50 different drug compounds with 646 ASDs were used for experiments and it was found that RF gave test set prediction accuracy of 82.5%, NB was the least accurate (46.67%), with all other models in the range of 70.83 to 80.83% [184]. The ANN modeling tool was used to achieve an enhanced dissolution rate of carbamazepine. The ternary solid dispersion of carbamazepine, Soluplus^®^, and Lutrol^®^ F68 was used as input parameters. The data for 32 experiments were generated using D-optimal mixture design and found *r*^2^ of 0.978 [136].

To predict Hansen solubility parameters from a dataset of 130 compounds, the simplified molecular input line entry system (SMILE) was used to generate connectivity features, indices, and physiochemical properties. This diverse collection of features served as input for multivariate adaptive training, which was extended to solvent–polymer miscibility prediction using binary classification and solvent-dependent drug-like solid dissolution models [188]. A linear regression (LR) model was used to predict the dispersion of potential of drug–polymer miscibility. The model inputs were molecular descriptor and 3D structural information which were derived from the molecular structure, topology and atomic properties of compounds. This method was applied to 12 different drug compounds with polymer PVP-VA64. The result found that root mean squared error of prediction ranged from 0.018 to 0.0782 [187]. An ANN and genetic programming (GP) model was developed to find the percentage drug release at 60 min, time required for 90% of the drug to dissolve, floating properties, and physical stability of the formulation. The model utilizes the input proportion of drug, polymer, and effervescent agent for 25 different mixtures. The result showed that univariate regression model (*logit P*(*Y*) = −1.927 + 0.208 T) with deviance of 6.513, and leave-one-out cross validation error of 0.3841 [186]. ANN was used to predict the percentage of the tibolone drug that dissolved within 30 min. The inputs for the model were the molecular weight of PEG, mixing temperature, amount of drug, and mixing time. The model was applied for thirty-six experiments and found that *r*^2^ is 0.9886, *p*-value < 0.001, and coefficient of variation is 3.53. The predictive ability of the optimal ANN structure was 0.048, indicating a strong correlation between examined factors and measured response [185].

**Figure 5:**
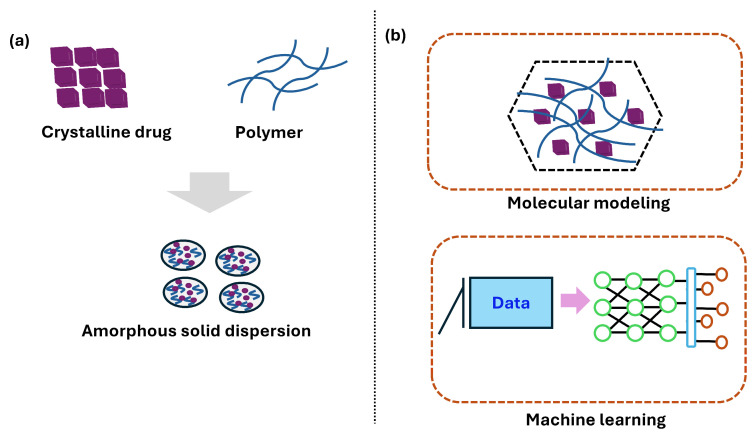
Schematic representation of (**a**) formation of amorphous solid dispersion of a poorly soluble drug in a carrier polymer, and (**b**) prediction of physical behavior, drug–polymer interaction, drug–polymer miscibility, and drug–polymer solubility using molecular modeling and machine learning.

Figure 5 illustrates that machine learning (ML) is increasingly valuable in addressing the long-term physical stability challenges of ASDs. Han et al. applied molecular modeling and eight distinct ML algorithms to predict ASD stability for three and six months. The random forest (RF) algorithm produced the most accurate model, identifying drug loading ratio, humidity, temperature, and molecular weight as critical stability determinants. This data-driven strategy improves formulation development efficiency and reduces dependence on empirical trial-and-error approaches [184]. The generalized regression (GR) neural network was employed to optimize the solid dispersion of carbamazepine with magnesium aluminosilicate (Neusilin^®^ UFL2) and Kollidon^®^ VA64. The ML approach yielded root mean square error (RMSE) values of 0.00029 and 0.1185 for the training and test data sets, respectively, indicating excellent predictive performance of the neural network [189]. ML models have also predicted chemically stable ASDs and identified critical attributes during manufacturing [183]. Additionally, a random forest regressor was used to predict the *T_g_* of polymers and drugs, providing insights into the impact of drug loading on *T_g_* and highlighting the importance of drug loading as a key feature in model training [182].

## 6. Future Perspectives

Physiologically based pharmacokinetic (PBPK) modeling is a robust tool for predicting drug absorption, distribution, metabolism, and excretion in humans. It integrates physiological and biochemical parameters to simulate drug behavior across populations, accounting for variations in sex, age, disease state, and metabolism [190,191]. PBPK modeling enables simulation of drug disposition in complex scenarios, potentially reducing reliance on human and animal trials. This approach has gained prominence in regulatory science, supporting drug development through evaluation of drug–drug interactions, first-in-human dosing, formulation design, and pharmacokinetics in special populations [190]. PBPK can incorporate the perturbed-chain statistical associating fluid theory (PC-SAFT) method to predict drug absorption and distribution. PC-SAFT is a thermodynamic model that estimates compound solubility and partitioning, which are essential for understanding drug interactions with biological systems. PBPK modeling has been applied to estimate drug solubility, partition coefficients, membrane permeability, drug distribution, and model interactions [192,193]. Studies have predicted pharmacokinetic profiles in rats and extrapolated findings to humans. In rats, pharmacokinetic analysis demonstrated higher C max and AUC, indicating enhanced absorption and brain penetration. PBPK model simulations corresponded with observed data, suggesting improved therapeutic efficacy in humans [194]. PC-SAFT predicts thermodynamic properties such as density, thermal expansion coefficient, glass transition temperature, isothermal compressibility, free energy change, and heat capacity. It is also used for phase equilibria calculations and chemical reaction predictions in ASD systems [195,196].

The emergence of novel artificial intelligence (AI)-based computational platforms, such as PharmSD, is transforming pharmaceutical development by advancing drug formulation and delivery processes through sophisticated computational methodologies. These platforms leverage ML and AI to analyze large datasets, optimize formulations, and predict drug interactions, thereby accelerating drug development and improving patient outcomes [197]. AI algorithms analyze formulation databases to identify optimal excipient combinations and processing parameters, substantially reducing experimental iterations. AI-driven process optimization ensures consistent product quality by implementing quality by design (QbD) principles. These systems utilize historical data to generate innovative formulation strategies and enhance drug efficacy [198]. It identifies the target by analyzing biological data to pinpoint disease-related targets, streamlining drug discovery, and increasing approval rates. Cost reduction is achieved through lead compound optimization, and AI minimizes the need for extensive animal testing [199]. The integration of AI with big data and the Internet of Things (IoT) delivers comprehensive solutions across the drug lifecycle, from discovery to registration [200]. These computational platforms employ various ML techniques, including RF, deep learning, support vector machines (SVMs), and gradient boosting algorithms, to predict drug dissolution profiles across different polymers, assess the physical stability of solid dispersions, and provide virtual screening tools to streamline formulation design [201].

Accelerated stability modeling techniques for predicting ASD stability primarily include the accelerated stability assessment program (ASAP) and advanced kinetic modeling (AKM). These approaches enable rapid and accurate prediction of drug stability, especially for ASD formulations prone to crystallization and chemical degradation. ASAP is a predictive stability modeling approach that uses accelerated temperature and humidity conditions to induce rapid degradation, followed by kinetic modeling to estimate shelf life under standard conditions [202]. The ASAPprime software operationalizes ASAP by guiding experimental design with ASAPdesign and modeling degradation kinetics to predict shelf life within one week instead of months or years [203]. It exposes products to multiple combinations of temperature (typically 50–70 °C) and humidity (10–80% RH), measuring the time-to-fail (isoconversion) at each condition [202]. ASAPprime then fits kinetic parameters such as activation energy (Ea), pre-exponential factor (ln A), and humidity sensitivity constant (B) using appropriate kinetic models, including diffusion kinetics, to accommodate the complex degradation behavior typical of ASDs [202]. AKM extends beyond traditional Arrhenius-based kinetics to capture degradation pathways and physical instabilities in ASDs, such as recrystallization and phase separation. It primarily assesses drug–polymer miscibility, drug–polymer interactions, manufacturing methods, and storage conditions, including temperature and humidity [204]. These models often incorporate multiparametric kinetic and probabilistic simulations to describe physical and chemical stability phenomena, enabling prediction of crystallization onset and other stability parameters within short experimental periods. AKM is particularly relevant for ASDs due to their complex microenvironment and the influence of excipients and moisture ingress during long-term storage [205]. This approach is less time- and resource-intensive than conventional testing, while enabling accelerated clinical supply and improved quality assurance [206].

## 7. Conclusions

ASDs represent an effective strategy to enhance the solubility and bioavailability of poorly soluble drugs, a persistent challenge in pharmaceutical formulation. This review thoroughly discusses the physical stability considerations highlighting both opportunities and challenges in their development. The main advantage of ASDs lies in maintaining the drug in a supersaturated state, thereby increasing the dissolution rate and therapeutic efficacy. However, this benefit is frequently compromised by physical instability, leading to phase separation and recrystallization. Thermodynamic, kinetic, and environmental factors are critical for ensuring long-term stability. The combination of QbD and PAT frameworks facilitates effective control over material and process attributes, enhancing product consistency. Recent advances in ML and molecular modeling further support prediction of drug–polymer interactions and formulation improvement, minimizing reliance on empirical approaches. Future directions include the integration of AI-driven predictive tools, PBPK modeling, and accelerated stability assessments to refine ASD formulation strategies. With the utilization of computational techniques with experimental validation, researchers can address remaining challenges in stability and clinical translation. ASDs continue to offer a robust and versatile approach for overcoming poor solubility and maintaining physical stability over time using predictive tools and ML.

## Figures and Tables

**Table 1:** Trends in amorphous solid dispersion drug products approved by the U.S. Food and Drug Administration (FDA) through 2023.

Manufacturing Method	Trade Name	Drug(s)	Polymer(s)	Dosage Form	Company	Year of Approval
Solvent evaporation	Cesamet^®^	Nabilone	PVP	Tablet	Valeant	1985
	Prograf^®^	Tacrolimus	HPMC	Capsule	Astella	1994
Fluidized bed layering	Sporanox^®^	Itraconazole	HPMC	Capsule	Janssen	1992
Spray drying	Crestor^®^	Rosuvastatin	HPMC	Tablet	AstraZeneca	2002
	Intelence^®^	Etravirine	HPMC	Tablet	Janssen	2008
	Samsca^®^	Tolvaptan	HPC	Tablet	Otsuka	2009
	Zortress^®^	Everolimus	HPMC	Tablet	Novartis	2010
	Incivek^®^	Telaprevir	HPMCAS	Tablet	Vertex	2011
	Kalydeco^®^	Ivacaftor	HPMCAS	Tablet	Vertex	2012
	Harvoni^®^	Ledipasvir/sofosbuvir	PVP-VA64	Tablet	Gilead	2014
	Epclusa^®^	Sofosbuvir/velpatasvir	PVP-VA64	Tablet	Gilead	2016
	Orkambi^®^	Lumacaftor/ivacaftor	HPMCAS	Tablet and granule	Vertex	2016
	Zepatier™	Elbasvir/grazoprevir	PVP-VA64	Tablet	Merck	2016
	Jynarque^®^	Tolvaptan	HPC	Tablet	Otsuka	2018
	Tibsovo^®^	Ivosidenib	HPMCAS	Tablet	Servier	2018
	Pifeltro^®^	Doravirine	HPMCAS	Tablet	Merck	2018
	Delstrigo^®^	Doravirine/lamivudine/tenofovir disoproxil fumarate	HPMCAS	Tablet	Merck	2018
	Tolsura^®^	Itraconazole	HPMCP	Capsule	Mayne	2018
	Erleada^®^	Apalutamide	HPMCAS	Tablet	Janssen	2018
	Symdeko^®^	Tezacaftor/ivacaftor and ivacaftor	HPMCAS	Tablet	Vertex	2018
	Trikafta^®^	Elexacaftor/ivacaftor/tezacaftor	HPMCAS	Tablet	Vertex	2019
	Qinlock^®^	Ripretinib	PVP-VA	Tablet	Deciphera	2020
	Sotyktu^®^	Deucravacitinib	HPMCAS	Tablet	Bristol	2022
	Sunlenca^®^	Lenacapavir	PVP-VA	Tablet	Gilead	2022
	Jaypirca^®^	Pirtobrutinib	HPMCAS	Tablet	Loxo Oncology	2023
Hot-melt extrusion	Isoptin^®^	Verapamil	HPC/HPMC	Tablet	Abbott	1987
	Rezulin^®^	Troglitazone	HPMC	Tablet	Pfizer	1997
	NuvaRing^®^	Etonogestrel and ethyl estradiol	EVA	Ring	Merck	2001
	Kaletra^®^	Ritonavir/lopinavir	PVP-VA64	Tablet	Abbott	2007
	Norvir^®^	Ritonavir	PVP-VA64	Tablet	Abbott	2010
	Onmel^®^	Itraconazole	HPMC	Tablet	Merz	2010
	Zelboraf^®^	Vemurafenib	HPMCAS	Tablet	Roche	2011
	Noxafil^®^	Posaconazole	HPMCAS	Tablet	Merck	2013
	Astagraf XL^®^	Tacrolimus	HPMC; EC	Capsule	Astella	2013
	Belsomra^®^	Suvorexant	PVP-VA64	Tablet	Merck	2014
	Viekira XR™	Dasabuvir/ombitasvir/paritaprevir/ritonavir	PVP-VA64; HPMC	Tablet	AbbVie	2014
	Venclexta^®^	Venetoclax	PVP-VA64	Tablet	AbbVie	2016
	Mavyret™	Glecaprevir/pibrentasvir	PVP-VA64	Tablet	AbbVie	2017
	Idhifa^®^	Enasidenib	HPMCAS	Tablet	Bristol	2017
	Lynparza^®^	Olaparib	PVP-VA	Tablet and capsule	AstraZeneca	2017
	Braftovi^®^	Encorafenib	PVP-VA64	Capsule	Array	2018
	Ubrelvy^®^	Ubrogepant	PVP-VA64	Tablet	AbbVie	2019
	Oriahnn^®^	Elagolix/estradiol/norethindrone acetate	PVP-VA	Tablet	AbbVie	2020
	Tukysa^®^	Tucatinib	PVP-VA	Tablet	Seagen	2020
	Xtandi^®^	Enzalutamide	HPMCAS	Tablet	Astella	2020
	Qulipta^®^	Atogepant	PVP-VA64	Tablet	AbbVie	2021
	Welireg^®^	Belzutifan	HPMCAS	Tablet	Merck	2021
	Paxlovid^®^	Nirmatrelvir/ritonavir	PVP-VA	Tablet	Pfizer	2023
	Alvaiz^®^	Eltrombopag	PVP-VA	Tablet	Teva	2023
Wet granulation	Orilissa^®^	Elagolix	HPMCAS	Tablet	AbbVie	2018
Electro spraying	Phyrago^®^	Dasatinib	Methacrylic acid-ethyl acrylate copolymer	Tablet	Nanocopoeia	2023

^®^ Registered trademark, ™ Trademark.

**Table 2:** Commonly used polymers and their physicochemical properties: category, molecular weight, *T_g_*, degradation temperature, hygroscopicity, solubility, and key features.

Category	Polymer Type	Polymer Subtype	Mol. wt. (g/mol)	*T_g_*/*T_m_* (°C)	Degradation Temp. (°C)	Moisture Retention	Solubility	Key Features	Reference
Cellulose derivative	HPMCAS	HPMCAS LG	144,700	119	204	Low	pH 5.5–6.0	Anionic	[44,45,54]
		HPMCAS MG	103,200	120	190	Low	pH 6.0–6.5	Anionic	[44,45,54]
		HPMCAS HG	75,100	122	200	Low	Above pH 6.8	Anionic	[44,45,54]
	HPMCP	HPMCP 50	37,900	137	160–190	Low	Below pH 5	Amphiphilic	[43]
		HPMCP 55	45,600	133	150	Low	below pH 5.5	Amphiphilic	[43]
	HPMC	HPMC E	40,000–150,000	141	NA	High	Water	Nonionic	[46]
		HPMC F	40,000–150,000	160	240	High	Water	Nonionic	[46]
		HPMC K	40,000–150,000	172	260	High	Water	Nonionic	[46]
		CAP	2534.12	175	200	Low	Below pH 6	Nonionic	[55]
Polyvinyl derivatives	PVP	PVP-K12	2000–3000	72	196	High	Water	Amphiphilic	[47,56]
		PVP-K17	7000–11,000	140	217	High	Water	Amphiphilic	[47,56]
		PVP-K25	28,000–34,000	153	166	High	Water	Amphiphilic	[47,56]
		PVP-K30	44,000–54,000	160	171	High	Water	Amphiphilic	[47,56]
		PVP-K90	1,000,000–1,500,000	177	194	High	Water	Amphiphilic	[47,56]
		PVP/VA	45,000–70,000	115	270	High	Water	Amphiphilic	[47,56]
		Soluplus^®^	90,000–140,000	72	278	Moderate	Water	Amphiphilic	[47,56]
Polymethacrylate derivatives	Eudragit^®^ EPO		47,000	48	250	Low	Below pH 5	Cationic	[48,49]
	Eudragit^®^ L100		125,000	150	176	Low	Above pH 6	Anionic	[48,49]
	Eudragit^®^ S100		125,000	>150	173	Low	Above pH 7	Anionic	[48,49]
	Eudragit^®^ L100-55		250,000	110	176	Low	Above pH 5.5	Anionic	[48,49]
Miscellaneous	PVAP		47,000–61,000	46/116	150	Low	Below pH 6	Nonionic	[50]
	PAA		1800–450,000	126	200	Low	Water	Nonionic	[51]
	PEG/POE		1000–7,000,000	55–66	>200	Low	Water	Nonionic	[52]
	Lutrol^®^		7600–17,400	52–57	>200	Low	Water	Nonionic	[53]

**Table 3:** Solvents used in preparation of ASD and their boiling point, solubility, density, viscosity, dielectric constant, and toxicity level according to ICH guidelines [59].

Solvent	Boiling Point	Solubility in Water (g/mL)	Density at 25 °C (g/mL)	Viscosity (at 25 °C, cP)	Dielectric Constant	ICH Class (Limit, ppm)
Acetone	56.2	Miscible	1.049	0.295	20.7	Class 3
Butanone	79.6	29	0.805	0.4	18.51	Class 3
Butyl acetate	126.1	0.68	0.882	0.685	5.07	Class 3
Chloroform	61.7	0.795	1.498	0.536	4.81	Class 2 (60)
Dichloromethane	39.6	1.32	1.326	0.413	9.08	Class 2 (600)
Dimethyl acetamide	165	Miscible	0.937	0.92	37.78	Class 2 (1090)
Dimethyl formamide	153	Miscible	0.944	0.97	36.7	Class 2 (880)
Dimethyl sulfoxide	189	25.3	1.092	1.987	47	Class 3
Ethanol	78.5	Miscible	0.789	1.04	24.6	Class 3
Ethyl acetate	77	8.7	0.895	0.428	6	Class 3
Glycerin	290	Miscible	1.261	954	42.5	-
Isopropanol	82.6	Miscible	0.786	1.96	18.2	Class 3
Methanol	64.6	Miscible	0.791	0.543	32.6	Class 2 (3000)
Tetrahydrofuran	66	Miscible	0.889	0.48	7.52	Class 2 (720)
Water	100	-	0.998	1	78.5	-

**Table 4:** Application of QMs, MMs, and docking studies in amorphous–solid dispersion systems to simulate drug–polymer interactions.

Drug(s)	Polymer(s)	Simulation	Software	Summary	Reference
Indomethacin	Eudragit^®^ PEO, glucose, sucrose	Molecular dynamics	Material Studio 4.0	Drug interaction with miscible (Eudragit^®^ PEO), immiscible (glucose), and low miscible polymer (sucrose).	[124]
Paclitaxel	PEG, PCL, MPEG-PCL	Molecular dynamics	HyperChem 8.0	Paclitaxel binds to MPEG–PCL copolymer, forming a core–shell structure.	[125]
Curcumin	MPEG-PCL	Molecular dynamics	HyperChem 8.0	An increased number of hydrophobic binding sites for curcumin improve stability and strong binding between copolymer and drug.	[126]
Artemisinin	PEG, PVP	Molecular dynamics	Material Studio 6.0	Polymers miscible with artemisinin, forming stable solid dispersions and suggesting molecular dispersion.	[127]
Lumefantrine	Soluplus^®^, Kollidon^®^ VA64, Plasdone™ S630	Molecular dynamics	Maestro Schrodinger 2025.1	Strong interactions occurred between hydroxyl and carbonyl groups of polymers and chlorine and amine groups of lumefantrine.	[128]
Cyclosporin A	L/D-polylactide, chitosan, polyglycolic acid, PEG, cellulose	Molecular docking	Materials Studio 6.0	Polycellulose and polychitosan exhibited high miscibility, due to large open surface.	[129]
Indomethacin	PVP	Molecular dynamics	AMBER24	Drug solubility increased with PVP dispersion.	[130]
Lafutidine	Soluplus^®^, PEG 400, Lutrol^®^ F127, Lutrol^®^ F68	Molecular dynamics	Maestro Schrodinger 2025.1	Interaction between polymer’s hydroxyl and carbonyl groups and drugs’s chlorine and amine group.	[131]
Posaconazole	Soluplus^®^, PEG 400, Lutrol^®^ F127, Lutrol^®^ F68, TPGS	Molecular dynamics	Maestro Schrodinger 2025.1	Hydrogen bonding between drug and polymer resulted in low energy and high binding interaction.	[132]
Propranolol HCl, diphenhydramine HCl, paracetamol, ibuprofen, diclofenac sodium, hydrocortisone	Eudragit^®^ L100, Eudragit^®^ EPO, Eudragit^®^ L100-55, Kollidon^®^ VA64	Quantum mechanical/DFT	Gaussian 09	Strength of interactions depended on donor and acceptor and number of hydrogen bonds between drug and polymer.	[133]
Cetirizine HCl, verapamil HCl	Eudragit^®^ L100, Eudragit^®^ L100-55	Molecular dynamics	Maestro Schrodinger 2025.1	Strong interactions between amine groups of drug and carboxylate groups of polymers, indicating high binding energy and stability.	[134]
Gemcitabine	Chitosan	Molecular dynamics	Material Studio 4.3	Drug loading from strong interaction between chitosan and drug.	[135]
Carbamazepine	Lutrol^®^ F68	Molecular dynamics	XenoView 3.8	Drug molecules showed strong tendency to aggregate.	[136]
Telaprevir	Cellulose derivatives	Quantum mechanical/DFT	HyperChem 8.0	Polymers contain carboxylate groups with optimal hydrocarbon chain length, resulting in favorable solvation free energy.	[137]
Tacrine	Chitosan, PBCA	Molecular dynamics	LAMMPS 2014	Interaction between tacrine and polymeric nanoparticles increased with polymer chain.	[138]
Indomethacin	PEG, PLA	Molecular dynamics	Material Studio 8.0	Drug miscibility with polymers, resulting in encapsulation efficiency.	[139]
Clonazepam, ibuprofen, fenofibrate, alprazolam	PVP-VA64, HPMC, Eudragit^®^ EPO	Molecular dynamics	Materials Studio 7.0	Ibuprofen/PVP-VA64 and ibuprofen/Eudragit^®^ EPO formed strong hydrogen bonds.	[140]
Felodipine	HPMC	Molecular dynamics	AMBER24	Polymer miscibility at various concentrations.	[141]
Aspirin, caffeine, carbamazepine, finasteride, flufenamic acid, flutamide, mefenamic acid, salicylamide, theophylline	PVP-VA64, poly (glycerol adipate) and derivatives	Molecular dynamics	GROMACS 5.1	Solubility and interaction parameters did not correlate with miscibility.	[142]
Ibuprofen, carbamazepine	Soluplus^®^, PEG	Molecular docking	AutoDock Vina 1.2.5	Ibuprofen-Soluplus^®^/PEG and carbamazepine-Soluplus^®^/PEG, with latter having strong interaction.	[143]
6-Mercaptopurine	PLA, PEG-modified PLA	Molecular docking	XenoView v.3.7.9.0	Polymerization degree was optimal for drug solubility in polymers.	[144]
Olmesartan medoxomil	PVP-VA64, Soluplus^®^	Molecular dynamics	Maestro Schrodinger 2025.1	Strong hydrogen bonding between carbonyl group of pyrrolidone and acetate monomers of PVP-VA64 and tetrazole and aromatic rings of olmesartan medoxomil inhibited recrystallization.	[145]
Simvastatin	PVP	Molecular dynamics	XenoView v.3.7.9.0	Simvastatin contains hydrogen bond donor and acceptor groups, while PVP contains hydrogen bond acceptors, resulting in intermolecular interactions and stabilization.	[146]
Rivaroxaban	Soluplus^®^	Molecular dynamics	XenoView v.3.7.9.0	Strong molecular interactions and Soluplus^®^ chain shrinkage led to recrystallization under high humidity.	[147]
Naproxen, indomethacin	PVP, PVA	Quantum mechanical/DFT	COSMO-SAC 2016	Drug–polymer solubility and thermodynamic compatibility study.	[148]
Ritonavir	Lutrol^®^	Molecular dynamics	GROMACS 5.1	Strong interactions suppressed molecular mobility and prevented recrystallization.	[22]
Erlotinib HCl	PEG, PVP	Molecular dynamics	Material Studio 7.0	Drug formed weak hydrogen bonds with individual polymers, while composite polymer enhanced molecular interactions.	[149]

**Table 5:** Recent machine learning prediction for amorphous solid dispersion properties, stability, and formulation.

Year	Target Feature	Input Feature	Algorithm	Dataset	Reference
2025	Dissolution kinetics	Molecular descriptors using various dissolution condition	LightGBM	616 dissolution profiles	[29]
2024	Morphological influence on solubility of drug	Spherical shape, diameter, and drug concentration	CNN	161 images	[180]
2024	Solubility and phase behavior	Crystalline API with polymers, HB interaction molecule descriptor	DNN	499 solubility data	[181]
2024	Determination of glass transition temperature determination (*T_g_*)	Hydrophilic backbone methylation, hydrophilic feed fraction, hydrophobic backbone methylation	RF	50 unique copolymers with probucol	[182]
2023	Amorphization and chemical stability of ASDs via HME	Proportions of drug and polymer, extruder configuration, barrel temperature, screw speed, and feed rate	XGBoost, Light GBM, RF, SVM, SHAP, IG	39 drug molecules	[183]
2020	Quantification and differentiation of amorphous solid dispersion systems	Crystalline and amorphous drug content of rivaroxaban with Soluplus^®^	ANN, PLS, PCR	30 sample formulations	[147]
2019	Physical stability of solid dispersions at 3 months and 6 months	Drug loading ratio, polymer molecular weight, drug properties, environmental conditions, preparation method, and temperature	ANN, SVM, RF, DT, LightGBM, kNN, NB, DNN	50 drug compounds with ten molecular descriptors	[184]
2015	Enhanced dissolution rate	Optimization of ternary solid dispersions of carbamazepine, Soluplus^®^, and Lutrol^®^ F68	ANN	22 using D-optimal mixture experimental design and three for predictive modeling	[136]
2013	The percentage of Tibolone dissolved in 30 min (Y30min)	Molecular weight of PEG, mixing temperature, drug amount, and total mixing time	ANN	36 experiments with four independent factors	[185]
2011	Percentage drug release at 60 min, time to 90% drug dissolution, floating properties, physical stability	Proportions of drug, polymer, and effervescent agents	ANN/GP	25 mixture proportions	[186]
2011	Dispersion potential of drug–polymer (miscible dispersion)	Molecular descriptors and 3D structure derived from molecular structure, topology, and atomic properties	LR	12 compounds solidified with PVP-VA64	[187]
